# Measuring quality of life in life-threatening illness – content validity and response processes of MQOL-E and QOLLTI-F in Swedish patients and family carers

**DOI:** 10.1186/s12904-020-00549-6

**Published:** 2020-03-25

**Authors:** Lena Axelsson, Anette Alvariza, Nina Carlsson, S. Robin Cohen, Richard Sawatzky, Kristofer Årestedt

**Affiliations:** 1 Department of Nursing Science, Sophiahemmet University, Box 5605, 114 86 Stockholm, Sweden; 2 Department of Health Care Sciences/ Palliative Research Centre, Ersta Sköndal Bräcke University College, Stockholm, Sweden; 3 Capio Palliative Care, Dalen hospital, Stockholm, Sweden; 4 Faculty of Health and Life Sciences, Linnaeus University, Kalmar, Sweden; 5 Departments of Oncology and Medicine, McGill University, Montreal, Quebec Canada; 6 Lady Davis Research Institute, Montreal, Quebec Canada; 7 School of Nursing, Trinity Western University, Langley, Canada; 8 Centre for Health Evaluation and Outcome Sciences, Providence HealthCare, St. Paul’s Hospital, Vancouver, Canada; 9 Sahlgrenska Academy University of Gothenburg, Gothenburg, Sweden; 10 The Research Section, Region Kalmar County, Kalmar, Sweden

**Keywords:** Patient, Family carer, Family caregiver, Instrument development, Response processes, Validity, Quality of life, Palliative care, End of life

## Abstract

**Background:**

The McGill Quality of Life Questionnaire - Expanded (MQOL-E) and the Quality of Life in Life-Threatening Illness-Family Carer/Caregiver version (QOLLTI-F) are developed for use with patients facing the end of life and their family carers, respectively. They are also developed for possible use as companion instruments. Contemporary measurement validity theory places emphasis on response processes, i.e. what people feel and think when responding to items. Response processes may be affected when measurement instruments are translated and adapted for use in different cultures. The aim of this study was to translate and examine content validity and response processes during completion of MQOL-E and QOLLTI-F version 2 (v2) among Swedish patients with life-threatening illness and their family carers.

**Methods:**

The study was conducted in two stages (I) translation and adaptation (II) examination of content validity and response processes using cognitive interviews with 15 patients and 9 family carers. Participants were recruited from the hemodialysis unit, heart clinic, lung clinic and specialized palliative care of a Swedish county hospital. Patients had life-threatening illness such as advanced heart failure, advanced chronic obstructive pulmonary disease, end-stage kidney disease or advanced cancer. Patients were outpatients, inpatients or receiving home care.

**Results:**

Patients and family carers respectively believed that the items of the MQOL-E and QOLLTI-F v2 reflect relevant and important areas of their quality of life. Although some items needed more time for reflection, both instruments were considered easy to understand. Some changes were made to resolve issues of translation. Participants expressed that reflecting on their situation while answering questions was valuable and meaningful to them, and that responding was an opportunity to express feelings.

**Conclusions:**

The results of response processes pertaining to the Swedish translations of both MQOL-E and QOLLTI-F v2 contribute evidence regarding content validity, linguistic equivalence and cultural appropriateness of the translated instruments. In addition, results show that the instruments may support conversations on matters of importance for quality of life between patients and/or family carers and health care professionals. Further research is needed to study the psychometric properties of Swedish translations.

## Background

The primary goal of palliative care is to enhance the quality of life (QOL) of people who have life-threatening illness and their families through the prevention and relief of suffering. This requires early identification of their concerns [1]. Hence there is a need to systematically assess both patients’ and family carers’ QOL [2]. Self-reported measurement instruments are increasingly used to identify physical, psychological, social and spiritual care needs, assess changes and evaluate interventions [3]. QOL measures developed for use in palliative care irrespective of the underlying diagnosis are few [4]. Although several disease specific instruments are available [2], there is a need for measures for use in palliative care irrespective of the disease.

The McGill Quality of life Questionnaire (MQOL) is a measure that was originally developed in Canada (in English and French) for patients with life-threatening illness (irrespective of specific disease) [5, 6]. It is now recommended and used internationally for palliative care irrespective of diagnosis [2, 4]. Developed for the specific situation when facing the end of life, MQOL incorporates assessment of existential well-being and includes not only negative but also positive contributors to QOL, while also considering the length of the instrument [5, 6]. This is important as measures should not be too burdening even though they need to comprise several aspects of QOL [7]. The increasing interest in self-reported measurement instruments for palliative care has resulted in the original MQOL being translated into about 20 languages. The measure has been recently revised and initially validated (MQOL-Revised; MQOL-R) [8]. The MQOL-R measures four domains: physical, psychological, existential, and social; it also includes an item assessing overall QOL. As the situation can change quickly at the end of life, it has a timeframe of 2 days. In addition, an expanded version has been developed, MQOL-Expanded (MQOL-E) [9], to include a wider variety of domains that people with life-limiting illness indicate are important to their QOL [10, 11]. MQOL-E incorporates MQOL-R and additional dimensions of QOL: feeling of being a burden, environment, cognition and quality of health care. It consists altogether of 21 items including the single item about overall QOL [9]. All items have a numeric response scale from 0 to 10, anchored with verbal responses at the ends/extremes. After reversed items have been rescored, zero describes the worst situation. An example of an item is “Over the past two days (48 hours) I felt: Physically terrible (0) vs. Physically well (10)” [12]. A summary of the item content for MQOL-E is presented in Table 1.

**Table 1:** Description of the McGill quality of life questionnaire - expanded (MQOL-E)

Item no.	Dimension	Item content
Single item	Overall QoL	Overall QoL
1	Physical	Problems with physical symptoms
2		Physical state
3		Problems due to physical functioning
4	Psychological	Being depressed
5		Being nervous or worried
6		Feeling sad
7		Fear of future
8	Existential	Meaning in life
9		Achievement of life goals
10		Control over life
11		Feeling about oneself
12	Social	Communication with people I care about
13		Relationships with people I care about
14		Feeling supported
15	Burden	Feeling about how one’s situation affects people I care about
16	Environment	Physical surroundings
17	Cognition	Clarity of thought
18		Memory function
19	Health care	Access to information
20		Availability of health care^a^
21		Quality of care

^a^Item 20 is excluded from the final published version of MQOL-E

The family carers’ situation is interwoven with the situation of the patient approaching the end of life [13, 14], and their QOL is affected as well. Hence the QOL of family carers should also be assessed. Furthermore, family carers are critical for care of the patient and home palliative care organization and they may need support [15]. The Quality of Life in Life-Threatening Illness - Family carer version (QOLLTI-F) is a companion instrument to MQOL-E and has been psychometrically validated with carers of cancer patients [16–18]. The QOLLTI-F originates from qualitative interviews exploring what family carers of people at the end of life indicate is important for their own QOL. The interview results identified not only burdens but also positive experiences in the caregiver’s situation which are included in the questionnaire. The QOLLTI-F includes seven subscales assessing different domains: environment, patient condition, the carer’s own state, carer’s outlook, quality of care, relationships and financial worries. The QOLLTI-F v2 consists of 17 items and, as in MQOL-E, a single item about overall QOL. Unique to QOLLTI-F is an item asking family carers about the patient’s condition, which is very important to their own QOL [17]. Like MQOL-E, each item has a numeric response scale ranging between 0 and 10, anchored with verbal responses at the ends/extremes and after reversed items have been rescored, zero describes the worst situation [17]. An example of an item is “Over the past two days (48 hours) I had time to take care of myself: Never (0) vs. Always (10)”. A summary of the item content for QOLLTI-F v2 is presented in Table 2. The QOLLTI-F has been translated into about 10 languages.

**Table 2:** Description of the quality of life in life-threatening illness - family carer/caregiver version (QOLLTI-F v2)

Item no.	Dimension	Item content
Single item	Overall QoL	Overall QoL
1	Environment	Satisfaction with place of care
2		Privacy
3	Patient condition	Distress related to patient condition
4	Carers own state	Control over life
5		Time to take care of oneself
6		Clarity of thought
7		Physical state
8		Emotional state
9	Carers outlook	Feeling about caring for the family member (patient)
10		Comfort from outlook, faith or spirituality
11		Meaning in life
12	Quality of care	Agreement with decision making process for patient
13		Availability of health care^a^
14		Quality of care
15	Relationships	Interaction with patient
16		Interaction with the other important people
17	Financial worries	Stress due to financial situation

^a^ Item 13 is excluded from the final version of QOLLTI-F v3

The availability of the instruments in different languages facilitates international comparisons. However, the validity of such comparisons requires that people are interpreting and responding to items of translated instruments in the same way (i.e., the translated items have the same meaning). To address this, contemporary measurement validity theory emphasizes examining response processes as a central aspect of measurement validity. Response processes describe “what people do, think, or feel when interacting with, and responding to, the item” [19]. Investigation of response processes and understanding how people interpret and respond to items is particularly important for determining whether the items and scores can be interpreted in the same way when measurement instruments are translated and adapted for use in different cultures. Evidence pertaining to content validity and response processes is essential to ensuring linguistic equivalence and cultural appropriateness of the items and validity of the overall measurement instrument [20]. Therefore, the aim of this study was to translate and examine content validity and response processes during completion of MQOL-E and QOLLTI-F v2 among Swedish patients with life-threatening illness and family carers.

## Methods

The study was conducted in two stages performed in the same way for each questionnaire: (I) translation and adaptation, and (II) examination of content validity and response processes among patients or family carers using cognitive interviews.

### Stage I: translation and adaption of the MQOL-E and QOLLTI-F v2

The translation of the MQOL-E and QOLLTI-F v2 from English to Swedish followed the European Organization for Research and Treatment of Cancer (EORTC) translation protocol [21]. First, the scales were translated into Swedish by two independent bilingual professional translators with Swedish as their native language. Thereafter the Swedish-speaking members of the research group discussed the translations and agreed on a first version for back translation by two independent professional translators who are native English speakers. The back translation was thereafter reviewed by one of the original MQOL-E and QOLLTI-F v2 creators (SRC) and critically discussed among the research group, for both linguistic and cultural aspects regarding all items. This thorough translation process for each instrument resulted in preliminary Swedish versions of MQOL-E and QOLLTI-F v2. 

### Stage II: content validity and response processes

Cognitive interviews [22, 23] using a think-aloud procedure together with probing questions were conducted with patients (MQOL-E) and family carers (QOLLTI-F v2) to evaluate content validity and response processes.

#### Participants and procedure

There were separate data collections for evaluation of MQOL-E and QOLLTI-F v2 which followed equivalent procedures. To be included in the study, participants had to be 18 years or older and able to read and understand Swedish. An additional inclusion criterion for the validation of MQOL-E was being a patient with life-threatening illness such as advanced heart failure (NYHA III-IV), advanced chronic obstructive pulmonary disease (COPD III-IV), end-stage kidney disease (CKD 5 and clinically assessed as possibly in their last year of life) or advanced cancer in specialized palliative care. For the validation of QOLLTI-F v2, participants had to be a family carer of a patient with such a life-threatening illness.

To gain varying perspectives purposive sampling [24] was used to achieve variation in age, gender, patient diagnosis and relationship between patients and family carers. The participants were recruited from the medical and geriatric departments in a county hospital in south-east Sweden including one hemodialysis unit, one heart clinic, one lung clinic and one specialized palliative care service. Altogether the settings included outpatient, inpatient and home care. A research nurse at each service/clinic identified and approached potential participants, i.e. patients or family carers, and provided verbal and written information about the study. One of the researchers contacted those who had shown interest and provided additional information and the opportunity to ask questions. Those who agreed to participate were asked for written informed consent at the time of the interview.

For the MQOL-E, 15 patients, 11 men and four women, aged 43–84 years (median = 72 years) participated. Three patients had a diagnosis of advanced heart failure, four had advanced chronic obstructive pulmonary disease, five had end-stage kidney disease and three had advanced cancer in specialized palliative care. Nine lived with a spouse or life partner. Their highest level of education varied between elementary school (*n* = 6), upper secondary school (*n* = 6) and university degree (*n* = 3).

For the QOLLTI-F v2, 9 family carers, four wives, one husband, one son, one daughter, a sister and a brother-in-law participated. Their ages ranged between 38 and 77 years (median = 64 years). One of the related patients had a diagnosis of advanced heart failure, two had advanced chronic obstructive pulmonary disease, two had end-stage kidney disease and four had advanced cancer in specialized palliative care. Family carers were all married, and their highest levels of education were elementary school (*n* = 3), upper secondary school (*n* = 4) and university degree (*n* = 2).

### Data collection and data analyses

Data was collected through cognitive interviews by two of the researchers between September 2016 and March 2017. The interviews were conducted using a think-aloud approach and probing questions [23] to explore participants’ understandings and reflections on the MQOL-E or QOLLTI-F v2 items concerning relevance, clarity, and sensitive content and wordings [23]. Before the interviews, participants were informed about the think aloud approach and encouraged to express their thoughts aloud and comment while completing all items in the questionnaire. They were also asked to convey their opinions regarding the clarity of items, response scale, relevance and content of the instrument, and length of the instrument (e.g. What do you think of the response alternatives with regard to the question? What do you think of the length of the instrument? Do you think that any question is redundant?). Participants were also asked if anything important was missing regarding aspects of QOL represented in the measurement instruments.

The cognitive interviews took place in a quiet room at the participant’s home or at the interviewer’s office at the university in accordance with the participant’s wishes. One patient and one family carer were interviewed by telephone. The interviews with patients lasted between 23 and 66 min (median = 33 min) while the interviews with family carers lasted 29–85 min (median = 57 min). All interviews except two with family carers were audio recorded and during all interviews the interviewer took field notes.

The analysis concerning content validity and response processes was based on Willis [23] recommendations to use overlapping approaches when analyzing cognitive interviews. The analysis of responses, pertaining to areas of relevance, clarity and sensitive content, was performed separately for each instrument. The analysis, regarding each item, began after the first interview to identify if rephrasing of any item was required, i.e., part of the analysis was concurrent with data collection. If rephrasing was required, the rephrased item was tested in subsequent interviews. Thereafter all recorded interviews were listened to (LA, NC) and analyzed together with interviewer notes (notes regarding e.g. observations of reactions and behavior taken during all interviews). This step of the analysis involved to identify content of responses related to areas of relevance, clarity and sensitive content. Next, the identified content (in interviews and notes) was summarized and coded pertaining to these areas (LA, NC). Thereafter text with codes were compared, discussed and sorted into pre-determined categories of relevance, clarity, and sensitive content and wordings (LA, NC, AA, KÅ).

## Results

### MQOL-E

#### Relevance

Patients thought that the overall instrument and items reflect relevant and important areas for their QOL. However, two patients who at the time of the interview were in a more stable stage suggested that the instrument would be more relevant later in the illness trajectory. One patient thought that the items about health care were irrelevant for QOL. Still, overall patients’ reflections indicated that the item of quality of health care was relevant and important for their perceived situation. Some patients thought that there should be a possibility to describe important matters to their QOL in open questions. For example, one patient thought that her daughter’s health influenced her response to the items. Others commented that they would like to express more about their worries for persons that are dependent on them, e.g. children. One patient suggested an item about how others’ behavior influenced his QOL, e.g. people at his children’s school or at his workplace. Furthermore, as the items were considered important and relevant to the patients’ situation the patients did not experience it as burdensome to answer, and the time needed was described as not a problem. When explicitly asked, MQOL-E was also believed to capture changes over time and hence worthwhile to answer on several occasions during the illness trajectory.

#### Clarity

The instructions were clear to all patients. During interviews some patients occasionally went back to the first page with the illustrating example for guidance. Most patients also said that the instrument was easy to understand and to answer. There were however comments that some items were unclear. Two patients commented that answers about if they “felt supported” depended on if it referred to health care professionals or family and friends. Two patients thought that “depressed” and “sad” were alike i.e. the same item while others answered these as two different items. Some patients needed more time to reflect over the item “control over life”, thinking of their serious disease but then associated the answer to the last 48 h. These comments did not lead to rewording as they were matters of reflection and interpretation rather than issues of translation.

Patients also commented on some of the verbal anchor responses as some found “terrified” when thinking of the future as too strong while one patient commented that “extremely good” was not a relevant answer concerning quality of health care, yet, another thought that it was just right.

The response scale of 0–10 was regarded as an advantage by some patients, as they were familiar with it from pain assessment scales, while others believed that a scale of 0–5 would be easier. Some also commented that in some scales the reverse verbal responses varied in strength. Between items in the instrument the response scale sometimes changes in direction and occasionally patients responded the reverse to what they intended.

#### Sensitive content and wordings

Patients did not consider any of the items upsetting or offensive, or that they evoked emotions that were hard to handle. However, about half of the patients showed various emotional reactions while responding. Their reactions varied from tears and sadness to expressing joy over parts of life. Still, none of them wanted to interrupt their completion of the MQOL-E or withdraw from the interview, as they thought it was important to participate. They expressed that reflecting over their situation while answering the items was valuable and meaningful to them. One patient expressed that it was difficult to answer the items about health care honestly when being dependent upon health care providers. Therefore, he suggested anonymous answers.

### QOLLTI-F v2

#### Relevance

Family carers thought that the overall instrument and items corresponded to their present situation and covered areas that are relevant and important for their QOL at this time. QOLLTI-F v2 was also proposed to be particularly relevant for carers of patients who are severely ill and cared for at home. One family carer thought that the item about finances was irrelevant but when reflecting over this she recognized its relevance. Yet, another commented that it was an important item. Some found the timeframe of 48 h problematic in relation to relevance; one because the last 48 h had been extreme, another because the time frame was, indeed, framing. In contrast, another family carer suggested that it was problematic as some items vary over hours, as, e.g., emotions.

There were suggestions to include items concerning support and information to the family from the health care professionals. One family carer suggested that an item about being tied up/feeling trapped should be added as the question about taking care of oneself was not considered the same. Also, an item that could capture the roller-coaster life as a carer was suggested.

As the instrument covered areas that were relevant and important to identify family carers’ situations and perspectives most family carers said that it was not strenuous to answer the items. Some also commented that it was quick to complete. When family carers’ opinions were requested, QOLLTI-F v2 was also believed to be worthwhile to fill in on several occasions during the illness trajectory to identify changes. The items also stimulated some family carers’ ideas for action, such as to initiate conversations with family members.

#### Clarity

The introductory instructions were clear to family carers. Overall the items were clear although some semantic unclarities were noted. For example, for the item “… I was satisfied with the place the family member/friend I’m caring for was staying (home, hospital, other)”, some family carers seemed to refer to the quality of care rather than the satisfaction with the place of care. In another item ( “… I had the privacy I wanted”) some seemed to think of private life as opposite work life rather than privacy. This resulted in a change of wording in the Swedish translation which resolved problems in clarity. One family carer found it unclear if satisfaction with providing care or company referred only to the patient or other family members which resulted in rephrasing. This clarified that the item concerns care of the patient. Family carers also suggested another Swedish wording for “stressful” concerning relations with the ill person. Hence this Swedish wording was also changed. One family carer commented that the item “take care of myself” needed reflection.

The response scale of 0–10 was regarded as clear and easy by most family carers, but suggested by others to be easier to use if it was narrowed to 0–5. The verbal response alternatives were regarded as in accordance with the questions. However, some family carers pointed out that the varying direction (negative or positive) of the response scale among items was tricky. They thought this demanded careful thinking. It was also questioned why the opposing responses were not equally strong/extreme in all items.

#### Sensitive content and wordings

No family carers thought that any item was upsetting or offensive, or evoked emotions that were hard to handle. However, when talking of their situation some carers’ eyes moistened with tears. One family carer suggested that if he had read the items beforehand, he might have been intimidated by all the personal questions. Yet, now he felt that reflecting over items and answers was important and rewarding. It came forth that reflecting over their own situation while answering the items was valuable to family carers.

Family carers suggested that having the opportunity to discuss responses with a health care professional would be of value. It was stressed as important that someone asked about their situation and that the use of QOLLTI-F v2 could be a way to be acknowledged by the health care professionals. Some family carers would prefer to answer the items at home and thereafter discuss their responses with a health care professional, while others thought that it would be better if a health care professional sat by their side while answering.

## Discussion

This study describes the translation into Swedish and subsequent initial validation of the content of two self-report companion instruments to measure QOL, MQOL-E for patients and QOLLTI-F v2 for family carers. Through the cognitive interviews, we examined response processes, i.e. how patients with different life-threatening illnesses and family carers interpret and respond to MQOL-E or QOLLTI-F v2 items while completing the translated instruments. Response processes are important as validity evidence to support the intended use of the measurement instruments and hence the validity of inferences in a new cultural context [19]. This is crucial as how people think and feel while responding to items may vary between different cultures, which may consequently influence the meaning of the scores.

The results of response processes pertaining to the Swedish translations of both MQOL-E and QOLLTI-F v2 contribute evidence regarding content validity, linguistic equivalence and cultural appropriateness of the translated instruments. After some changes to the translation based on the initial cognitive interviews, the items were understood and interpreted as intended and the instruments were appreciated as relevant and important to the target groups. The items triggered reflections in areas of importance and participants emphasized that responding was meaningful to them.

The results from the cognitive interviews provided important insights on the translation, which made it possible to correct and improve the clarity of the Swedish versions. The analysis showed that the issues were questions of choosing the optimal word for clarity in the translation rather than cultural differences. The results also show that the content was generally considered relevant for both MQOL-E and QOLLTI-F v2. This may be related to the fact that qualitative interviews were the foundation for both instruments, which were developed from patients’ or family carers’ views on what is important to them towards end of life [6, 17].

Two patients who at the time were in a more stable stage perceived that the MQOL-E would have increased relevance later in their illness trajectory. QOLLTI-F v2 was also proposed to be particularly relevant for carers of severely ill patients in homecare. This suggests that the instruments may be more relevant in late rather than early palliative care, but results also indicate that respondents felt that for understanding changes in their QOL over time (or as the disease progresses), it is important to include these items at earlier as well as later stages.

The patients’ suggestions for additional items needed to capture QOL in MQOL-E concerned different aspects of other persons’ influence on QOL. This highlights the significance of people in the environment and social interaction towards end of life. The original MQOL included an item on “the world has been: an impersonal, unfeeling place vs. caring and responsive to my needs” but was taken out in favor of an item referring to “people I care about …” because “the world” was unclear to some people.

Some family carers suggested that QOLLTI-F v2 is missing items on some aspects of being a family carer. One suggested a specific question on information from health care professionals to the family. This is in line with studies showing that to know what to expect in the future is a major need in family carers [25–27].

Both for MQOL-E and QOLLTI-F v2 the suggested additional items illuminate that QOL is an individual construct and that different aspects vary in importance [28]. However, in general the participants found the instruments relevant and the content important and engaging. In instrument development there are always decisions on what is most important versus the number of items and respondent burden [29]. In clinical care, one way to handle items suggested as additions without expanding the instruments might be to make place for the possibility to comment in free text on the content of the instrument [30].

Some family carers found that the timeframe of 48 h in QOLLTI-F v2 was problematic. Interestingly, these were in different ways i.e. both as too short and framing and as too long since some items vary over hours. This highlights the instability of some aspects of QOL in life-threatening illness and is important to consider when assessing it. This variability towards the end of life was also the reason why the original developer decided on a 48-h timeframe for the responses in MQOL and QOLLTI-F, rather than the more common timeframes of a week or a month [6, 17]. However, it is important to bear in mind that the last 48 h may have been extreme in some sense and scores should not be interpreted as representative of QOL over a longer time.

There were some comments that it would be easier if the response scale was narrowed to 0–5. However, in development of these measures it was found that a scale of 0–10 was accepted as intuitively easy by most participants (Cohen, personal communication) which was also the case in the present study. There were also some comments that the opposing end anchors varied in strength. However, this was purposefully done to get a decent distribution for some items, as in the development of both the MQOL and the QOLLTI-F, no one selected responses near the extreme negative end anchor (Cohen, personal communication).

The present results show that some patients occasionally responded reverse to what they intended in the items where the response scale changes the direction. This was also pointed out by family carers as tricky and demanded attention before responding. This issue is however complex and not easy to avoid as people have different ways of thinking of “10”: either as “the most” (whether or a positive or negative thing) or as “the best”. The consequence of changing the positive or negative direction in response scales has been studied before but it was found that there was not enough reason to change this as several factors influence answers [31]. Therefore, given the intent to retain comparability with the original instrument, the directions of the response scales remained unaltered in the Swedish translated versions.

When reflecting over items several patients and some family carers showed emotional reactions. These results show that the contents of MQOL-E and QOLLTI-F v2 are important and central to respondents and that responding may be an opportunity to reflect over important issues/questions and to express feelings as sadness but also joy. These results also show that MQOL-E and QOLLTI-F v2 may support meaningful conversations on aspects of QOL between patients and/or family carers and health care professionals. The use of QOLLTI-F v2 could be a way for family carers to communicate their own situation and be acknowledged by the health care professionals, which are aspects fundamental in palliative care. Importantly, studies have also shown that vulnerable groups such as patients at end of life [32] and their family members [33, 34] may find it valuable and important to participate in research.

### Methodological considerations

Patients had different life-threatening illnesses such as advanced heart failure, advanced chronic obstructive pulmonary disease, end-stage kidney disease or advanced cancer and both patients and family caregivers were recruited from different settings (inpatient, outpatient and home care) which add to the strength of the study. In studies using cognitive interviews the number of interviews needed varies and depends on the objective and the data quality [23]. Purposive sampling was used to achieve variation in age, gender, education and relationships, which added richness to the data. Furthermore, data in interviews was repetitive. Hence, the number of participants was considered sufficient to investigate content validity and response processes. Nevertheless, it is always possible that eligible participants that declined participation may have had different perspectives from the ones included. The description of background characteristics of the actual sample and the findings should facilitate judgement of transferability of findings to other contexts.

The patients were in both more stable phases and at the end of life. The patients that were interviewed about the MQOL-E were however not in the very end of life (as within a few days or weeks). This may influence the results as their views on QOL items may be related to the stage of their illness. While participants thought that the measures would capture change over time, we do not know in fact that they do in Sweden. Further validity evidence is needed to confirm psychometric equivalence, i.e. measurement invariance, of the translated and original versions of the instruments, and to ascertain sensitivity in measuring change over time [35].

In the present study we translated and validated an interim version of MQOL-E and the version 2 of QOLLTI-F. During the study a validated version of MQOL-E has been published and item 20 (Availability of health care) has been excluded. No other changes were made [9]. Furthermore, a version 3 of QOLLTI-F has been developed. The only change that has been made from QOLLTI-F v2 is that item 13 (Availability of health care) has been excluded. The present validation is therefore significant also for the latest versions of the instruments^1^.

## Conclusions

This study contributes important validity evidence about the response processes pertaining to both MQOL-E and QOLLTI-F v2 respectively, specifically with respect to linguistic equivalence and cultural appropriateness of the Swedish translated instruments. Findings demonstrate the importance of exploring and considering response processes in instrument development and translations in different languages and cultures. Psychometric studies of the Swedish translations of MQOL-E and QOLLTI-F are required before a firm recommendation can be made for their use in end-of-life and palliative care. The instruments are developed to be used irrespective of underlying diagnosis, and for possible use in conjunction, which increases the potential of their utilization in both clinical practice and research.

## Data Availability

The datasets generated and analysed during the current study are not publicly available due to ethical regulations but are available from the corresponding author on reasonable request.

## Abbreviations

MQOL-E: McGill quality of life questionnaire- expanded
QOLLTI-F: Quality of life in life-threatening illness - family carer/caregiver version
QOL: Quality of life

## Footnotes

1 The questionnaires can be obtained from robin.cohen@mcgill.ca and in Swedish also from kristofer.arestedt@lnu.se
